# Examining Non-Linear Associations between Accelerometer-Measured Physical Activity, Sedentary Behavior, and All-Cause Mortality Using Segmented Cox Regression

**DOI:** 10.3389/fphys.2016.00272

**Published:** 2016-06-29

**Authors:** Paul H. Lee

**Affiliations:** School of Nursing, Hong Kong Polytechnic UniversityHong Kong, China

**Keywords:** dose-response relationship, exercise, public health, restricted cubic spline, sitting, survival analysis

## Abstract

Healthy adults are advised to perform at least 150 min of moderate-intensity physical activity weekly, but this advice is based on studies using self-reports of questionable validity. This study examined the dose-response relationship of accelerometer-measured physical activity and sedentary behaviors on all-cause mortality using segmented Cox regression to empirically determine the break-points of the dose-response relationship. Data from 7006 adult participants aged 18 or above in the National Health and Nutrition Examination Survey waves 2003–2004 and 2005–2006 were included in the analysis and linked with death certificate data using a probabilistic matching approach in the National Death Index through December 31, 2011. Physical activity and sedentary behavior were measured using ActiGraph model 7164 accelerometer over the right hip for 7 consecutive days. Each minute with accelerometer count <100; 1952–5724; and ≥5725 were classified as sedentary, moderate-intensity physical activity, and vigorous-intensity physical activity, respectively. Segmented Cox regression was used to estimate the hazard ratio (HR) of time spent in sedentary behaviors, moderate-intensity physical activity, and vigorous-intensity physical activity and all-cause mortality, adjusted for demographic characteristics, health behaviors, and health conditions. Data were analyzed in 2016. During 47,119 person-year of follow-up, 608 deaths occurred. Each additional hour per day of sedentary behaviors was associated with a HR of 1.15 (95% CI 1.01, 1.31) among participants who spend at least 10.9 h per day on sedentary behaviors, and each additional minute per day spent on moderate-intensity physical activity was associated with a HR of 0.94 (95% CI 0.91, 0.96) among participants with daily moderate-intensity physical activity ≤14.1 min. Associations of moderate physical activity and sedentary behaviors on all-cause mortality were independent of each other. To conclude, evidence from this study supported at least 15 min per day of moderate-intensity physical activity and no more than 10.9 h per day of sedentary behaviors as recommendations to reduce all-cause mortality.

## Introduction

There is ample research showing that lack of physical activity and increased time spent in sedentary behaviors are detrimental to health and will increase mortality risk. Moderate-to-vigorous physical activity is defined as activities with energy expenditure of more than 3.0 metabolic-equivalent tasks (METs) and sedentary behaviors are defined by the Sedentary Behavior Research Network (Sedentary Behaviour Research Network, 2012) as “any waking behavior characterized by an energy expenditure of 1.5 or less METs while in a sitting or reclining posture.” Independent of physical activity, sedentary behaviors are detrimental to our health and will increase the risk of mortality as well (Biswas et al., 2015). Given the evidence of the health benefits of adequate physical activity, the American Heart Association and the American College of Sports Medicine jointly published a physical activity recommendation that healthy adults engage in at least 150 min per week (30 min per day for 5 days each week) of moderate-intensity physical activity (3.0–5.9 METs) or 60 min per week (20 min per day for 3 days each week) of vigorous-intensity physical activity (≥6.0 METs) (Haskell et al., 2007). A recent review have shown that there are many interventions promoting physical activity in the primary care setting according to this recommendation (Orrow et al., 2012).

The aforementioned physical activity recommendations were supported by studies using self-reported physical activity, for which validity is questionable (Van Poppel et al., 2010; Lee et al., 2011). In recent years, accelerometers are becoming more popular due to their good validity and reliability in measuring both physical activity (Plasqui and Westerterp, 2007) and sedentary behaviors (Kozey-Keadle et al., 2011). Many studies have examined the relationship of accelerometer-measured physical activity and sedentary behaviors to health outcomes and found that these associations differed from those based on self-reported (Lee and Wong, 2015). There have been several studies examining accelerometer-measured physical activity and sedentary behaviors on all-cause mortality, what we know was that accelerometer-measured physical activity was negatively associated with all-cause mortality among diabetic patients (Loprinzi, 2016) and those aged 50 or above (Schmid et al., 2015, 2016; Fishman et al., 2016). All these studies assumed that time spent in physical activity and sedentary behaviors were linearly associated with all-cause mortality, ignoring the possibility of “diminishing marginal returns” that continuously spending time in physical activity may decrease the benefits to health at some point. The aims of this study are to undertake such an examination in a representative US sample of general population and to examine such non-linear association, as well as the interaction effect between accelerometer-measured physical activity and sedentary behaviors, on all-cause mortality, using segmented Cox regression to empirically determine the break-points of the dose-response relationship.

## Materials and methods

### Participants

This study utilized data collected from participants in the National Health and Nutrition Examination Survey (NHANES) waves 2003–2004 and 2005–2006. The data are publicly available from the study website (http://www.cdc.gov/nchs/nhanes/about_nhanes.htm). The sampling method involved a multi-stage probability cluster design, and the sample was representative of the United States population. Participants were invited to complete a survey and a health examination; details of the instrument can be obtained from the NHANES website (http://www.cdc.gov/nchs/nhanes/nhanes_questionnaires.htm). Only data from adults aged 18 or above were analyzed in the present study. All participants aged 85 and over were coded as 85 to protect privacy. Consent was obtained from all participants, and the study was approved by the Centers for Disease Control and Prevention's ethics review board.

### Mortality

The NHANES data were linked using a probabilistic matching approach with death certificate data in the National Death Index through December 31, 2011. Mortality was classified according to the 10th Revision of International Classification of Diseases (ICD-10). On average, participants had been followed for 6.7 (SD 1.4) years. Participants with no mortality status were assumed to be alive.

### Accelerometer

All participants aged 6 and above were invited to wear an ActiGraph model 7164 (ActiGraph LLC) accelerometer over the right hip for 7 consecutive days at all times except when bathing or sleeping. The accelerometer data were recorded in 1-minute epochs. Following the data processing guidelines (http://www.riskfactor.cancer.gov/tools/nhanes_pam), accelerometer data that were unreliable were discarded. Each minute with accelerometer count <100; 100–1951; 1952–5724; and ≥5725 were classified as sedentary, light activity, moderate-intensity physical activity, and vigorous-intensity physical activity, respectively (Freedson et al., 1998). Daily wearing time was defined as 24 h minus non-wearing time; while non-wearing time was defined as an interval of zero counts for 60 consecutive minutes or more. A day was considered valid if it included at least 10 h of accelerometer-wearing time. Out of the 11,183 adult participants from the NHANES 2003–2006 studies, 9239 wore an accelerometer. Of these, 7088 provided at least 4 valid days of accelerometer data; to remove possible confounding factor those who were deceased within 12 months of the follow-up period were excluded (*n* = 82, 1.2%), leaving a final sample of 7006.

### Measurement

At the mobile examination center, blood pressure of participants was measured by certified blood pressure examiners. Blood pressure was measured three times (sometimes four), and the mean values were used. Participants were classified as having high blood pressure if they reported having doctor-prescribed medication for hypertension or had a blood pressure of ≥140/90 mmHg. Height and weight were measured by trained interviewers and BMI was calculated as weight (kg) divided by the squared height (m^2^). Participants were classified as overweight if they had BMI >25 kg/m^2^.

A blood test was conducted at the mobile examination center to obtain the levels of fasting plasma glucose and cholesterol (total, high-density lipoprotein (HDL), low-density lipoprotein (LDL), and triglycerides). Participants were classified as having type 2 diabetes if they either reported having doctor-prescribed medication for type 2 diabetes, or had fasting plasma glucose >126 mg/dL or glycated hemoglobin >6.5%. Participants were classified as having high cholesterol if they either reported having doctor-prescribed medication for high cholesterol, or had LDL cholesterol >130 mg/dL.

### Statistical analysis

Data were analyzed in 2016. Cox regression was used to estimate the hazard ratio (HR) of time spent in sedentary behaviors, moderate-intensity physical activity, and vigorous-intensity physical activity to all-cause mortality. Light activity was not included in the regression as the association between time spent in light activity and all-cause mortality as light activity was strongly correlated with sedentary behaviors (−0.51). The dose-response associations were initially examined with a restricted cubic spline model with 4 knots. In the final model, variables showing only linear associations with all-cause mortality were replaced by a linear term, and variables showing non-linear associations with all-cause mortality were analyzed by segmented regression with break-points estimated (Muggeo, 2003). The HR was adjusted for age, sex, education level, income, BMI, binge drinking, smoking status, energy intake by 24-h dietary recall (Moshfegh et al., 2003), self-reported general health condition, high blood pressure, high cholesterol, type 2 diabetes, and history of heart attack, stroke, and cancer. Race/ethnicity was not adjusted as it was not associated with time spent sedentary behaviors (Matthews et al., 2008). *Post-hoc* analysis of interaction effect between moderate physical activity and sedentary behaviors on all-cause mortality was conducted by fitting Cox regression with a restricted cubic spline model among subgroups with different levels of moderate physical activity and sedentary behaviors. Isotemporal models were used to estimate the association of substituting 1 min of sedentary behavior by 1 min of light activity, moderate-intensity physical activity or vigorous-intensity physical activity, while holding time spent in other activities constant (Mekary et al., 2009). All statistical analyses were conducted using R 3.2.0. The restricted cubic spline was fitted using package *rms* (Durrleman and Simon, 2006), the segmented regression was fitted using package *segmented* (Muggeo, 2008), and all confounders with missing data were imputed using the additive regression in package *Hmisc*.

## Results

### Characteristics of participants and patterns of objectively-measured sedentary behaviors and physical activity

On average, participants spent 9.3 h, 22.2 min, and 1.4 min on sedentary behaviors, moderate-intensity physical activity, and vigorous-intensity physical activity per day, respectively. Time spent in moderate- and vigorous-intensity physical activity per day were positively skewed, with 3776 (53.9%) participants not engaging in any of the latter type of activity. During the 47,119 person-year follow-up period, 608 deaths were observed. Table 1 shows that, greater physical activity was associated with survivors, male sex, younger age, better self-reported general health condition, no history of heart attack, stroke, or cancer, never smoking, higher family income, non-overweight, and absence of high blood pressure, high cholesterol, or type 2 diabetes.

**Table 1:** **Descriptive statistics**.

**Variable**	**N (%)**	**Mean hour spent sedentary behaviors per day (SD)**	**Mean minute spent moderate-intensity physical activity per day (SD)**	**Mean minute spent vigorous-intensity physical activity per day (SD)**
All participants	7006 (100%)	9.3 (2.3)	22.2 (21.9)	1.4 (7.5)
**MORTALITY**				
Survived	6398 (91.3%)	9.1 (2.2)^***^	23.5 (22.1)^***^	1.5 (7.8)^***^
Deceased	608 (8.7%)	10.9 (3.0)	8.8 (13.3)	0.2 (0.8)
**GENDER**								
Male	3400 (48.5%)	9.4 (2.5)^**^	28.3 (25.7)^***^	1.8 (8.7)^***^
Female	3606 (51.5%)	9.2 (2.2)	16.4 (15.5)	1.0 (6.2)
**AGE**								
18–29	1621 (23.1%)	8.9 (2.1)^***^	30.7 (24.4)^***^	2.2 (8.2)^***^
30–39	1040 (14.8%)	8.5 (2.1)	28.6 (22.8)	1.4 (6.9)
40–49	1103 (15.7%)	8.8 (2.1)	27.7 (23.8)	1.7 (6.8)
50–59	898 (12.8%)	9.2 (2.2)	21.1 (18.6)	1.0 (5.2)
60–69	1063 (15.2%)	9.5 (2.3)	15.2 (15.6)	1.0 (9.3)
≥70	1281 (18.3%)	10.7 (2.6)	8.1 (11.1)	0.6 (7.2)
**BINGE DRINKER**				
Yes	905 (12.9%)	9.3 (2.6)	23.2 (22.6)	1.3 (8.4)
No	6101 (87.1%)	9.3 (2.3)	22.1 (21.8)	1.4 (7.4)
**SELF-REPORT GENERAL HEALTH CONDITION**				
Excellent	734 (10.5%)	9.2 (2.2)^***^	28.8 (23.6)^***^	2.2 (5.2)^***^
Very good	2164 (30.9%)	9.2 (2.2)	24.6 (21.9)	1.7 (7.8)
Good	2681 (38.3%)	9.3 (2.3)	21.3 (21.4)	1.1 (6.3)
Fair	1225 (17.5%)	9.3 (2.6)	17.8 (21.2)	1.3 (10.6)
Poor	202 (2.9%)	10.0 (2.8)	10.6 (13.1)	0.2 (0.8)
**HISTORY OF HEART ATTACK**				
Yes	298 (4.3%)	10.6 (2.4)^***^	9.7 (12.7)^***^	0.9 (10.0)
No	6708 (95.7%)	9.2 (2.3)	22.8 (22.1)	1.4 (7.4)
**HISTORY OF STROKE**				
Yes	233 (3.3%)	10.7 (2.7)^***^	9.1 (12.7)^***^	0.8 (6.1)
No	6773 (96.7%)	9.2 (2.3)	22.7 (22.0)	1.4 (7.5)
**HISTORY OF CANCER**				
Yes	598 (8.5%)	10.1 (2.4)^***^	12.6 (14.7)^***^	1.6 (12.1)
No	6408 (91.5%)	9.2 (2.3)	23.1 (22.2)	1.4 (6.9)
**EVER SMOKER**				
Yes	3304 (47.2%)	9.4 (2.5)^**^	22.1 (22.5)	1.2 (7.2)
No	3702 (52.8%)	9.2 (2.2)	22.3 (21.4)	1.5 (7.8)
**ANNUAL FAMILY INCOME**				
<US20000	2041 (29.1%)	9.5 (2.6)^***^	21.1 (23.3)^**^	1.1 (5.3)
≥US20000	4965 (70.9%)	9.2 (2.2)	22.7 (21.3)	1.5 (8.2)
**COLLEGE GRADUATE**				
Yes	1313 (18.7%)	9.2 (2.4)^***^	21.9 (22.6)^*^	1.3 (7.7)^**^
No	5693 (81.3%)	9.8 (2.1)	23.5 (18.3)	2.0 (6.3)
**OVERWEIGHT**				
Yes	4674 (66.7%)	9.3 (2.3)	20.5 (20.6)^***^	1.2 (8.1)^**^
No	2332 (33.3%)	9.2 (2.4)	25.5 (23.9)	1.7 (6.1)
**HIGH BLOOD PRESSURE**				
Yes	2548 (36.4%)	9.9 (2.4)^***^	14.3 (17.4)^***^	0.8 (6.7)^***^
No	4458 (63.6%)	9.0 (2.2)	26.7 (22.9)	1.7 (7.9)
**HIGH CHOLESTEROL**				
Yes	3255 (46.5%)	9.6 (2.4)^***^	17.3 (18.7)^***^	0.9 (6.6)^***^
No	3751 (53.5%)	9.1 (2.2)	26.5 (23.5)	1.8 (8.2)
**TYPE 2 DIABETES**				
Yes	1117 (46.5%)	9.9 (2.4)^***^	13.1 (16.1)^***^	0.8 (8.4)^**^
No	3751 (53.5%)	9.2 (2.3)	23.9 (22.4)	1.5 (7.3)

*, **, *** *significant at 5, 1, and 0.1% level*.

### Dose-response relationship between objectively-measured sedentary behaviors and physical activity on all-cause mortality

**Figure 1:**
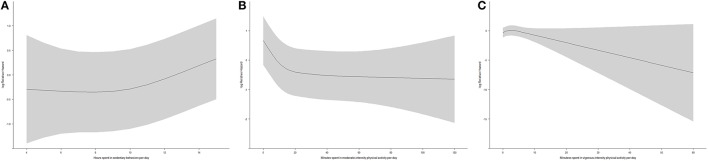
**(A)** The dose-response relationship between time spent in sedentary behaviors and all-cause mortality from restricted cubic spline regression (*N* = 7006, shaded area: 95% confidence interval); **(B)** The dose-response relationship between time spent in moderate-intensity physical activity and all-cause mortality from restricted cubic spline regression (*N* = 7006, shaded area: 95% confidence interval); **(C)** The dose-response relationship between time spent in vigorous-intensity physical activity and all-cause mortality from restricted cubic spline regression (*N* = 7006, shaded area: 95% confidence interval).

Figure 1 shows the dose-response relationships between time spent in each type of activity and all-cause mortality. Figure 1A shows that the risk of all-cause mortality for 4–9 h spent in sedentary behaviors per day remained constant, and that the risk increased linearly when daily sedentary behaviors exceeded 9 h per day. Figure 1B shows that the risk of all-cause mortality decreased with time spent in moderate-intensity physical activity. A reverse J-shaped association was observed, with the turning point at about 15 min per day. Figure 1C shows that the risk of all-cause mortality decreased with time spent in vigorous-intensity physical activity in a linear manner.

**Figure 2:**
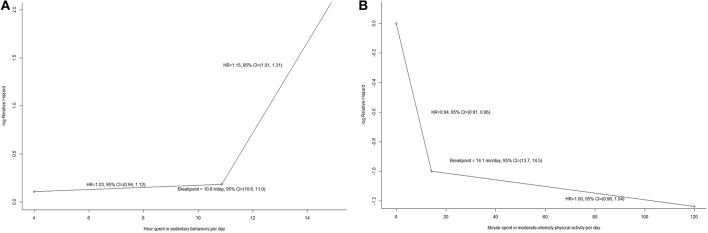
**(A)** The relationship between time spent in sedentary behaviors and all-cause mortality from segmented regression (*N* = 7006); **(B)** The relationship between time spent in moderate-intensity physical activity and all-cause mortality from segmented regression (*N* = 7006).

In accordance with the aforementioned results, the daily minutes spent in vigorous-intensity physical activity in the Cox regression model were replaced with their linear terms, and the daily sedentary hours and daily minutes spent in moderate-intensity physical activity per day were analyzed by a segmented Cox regression with one breaking point to form the final model. Figure 2 shows the log relative hazard yielded from the segmented Cox regression. The fitted breakpoint for the daily sedentary hours and daily moderate-intensity physical activity minutes were 10.9 (SE 0.1) and 14.1 (SE 0.2), respectively. Each additional sedentary hour per day was associated with a HR of 1.15 (95% CI: 1.01, 1.31, *P* = 0.03) among participants who had at least 10.9 h per day of sedentary behaviors. Among participants who spent at most 14.1 min per day on moderate-intensity physical activity, each additional minute spent such activity per day was associated with a HR of 0.94 (95% CI: 0.91, 0.96, *P* < 0.001), whilst among participants who spent more than 14.1 min per day, each additional minute spent such activity per day was not associated with all-cause mortality (HR: 1.00, 95% CI: 0.96, 1.04, *P* = 0.91). Each additional minute spent vigorous-intensity physical activity per day was associated with an insignificant HR of 0.96 (95% CI: 0.90, 1.02, *P* = 0.19).

**Figure 3:**
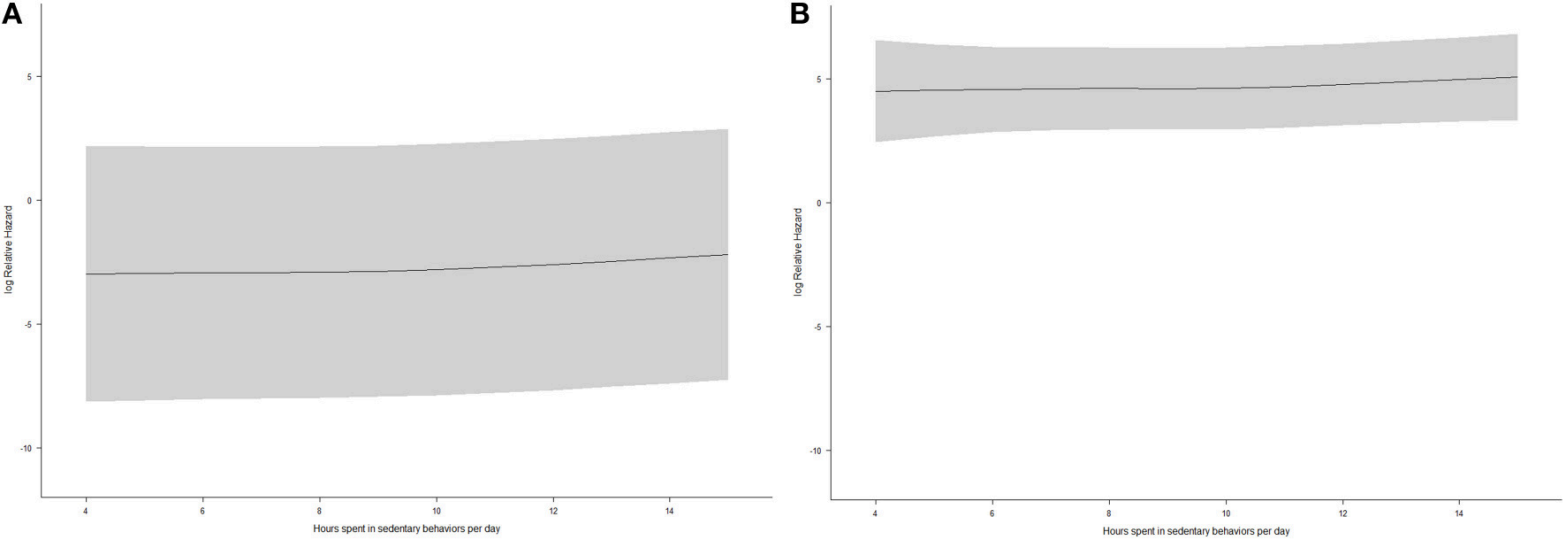
**(A)** The dose-response relationship between time spent in sedentary behaviors and all-cause mortality from restricted cubic spline regression (time spent in moderate physical activity per day ≤14.1 min, *N* = 3189, shaded area: 95% confidence interval); **(B)** The dose-response relationship between time spent in sedentary behaviors and all-cause mortality from restricted cubic spline regression (time spent in moderate physical activity per day >14.1 min, *N* = 3817, shaded area: 95% confidence interval).

**Figure 4:**
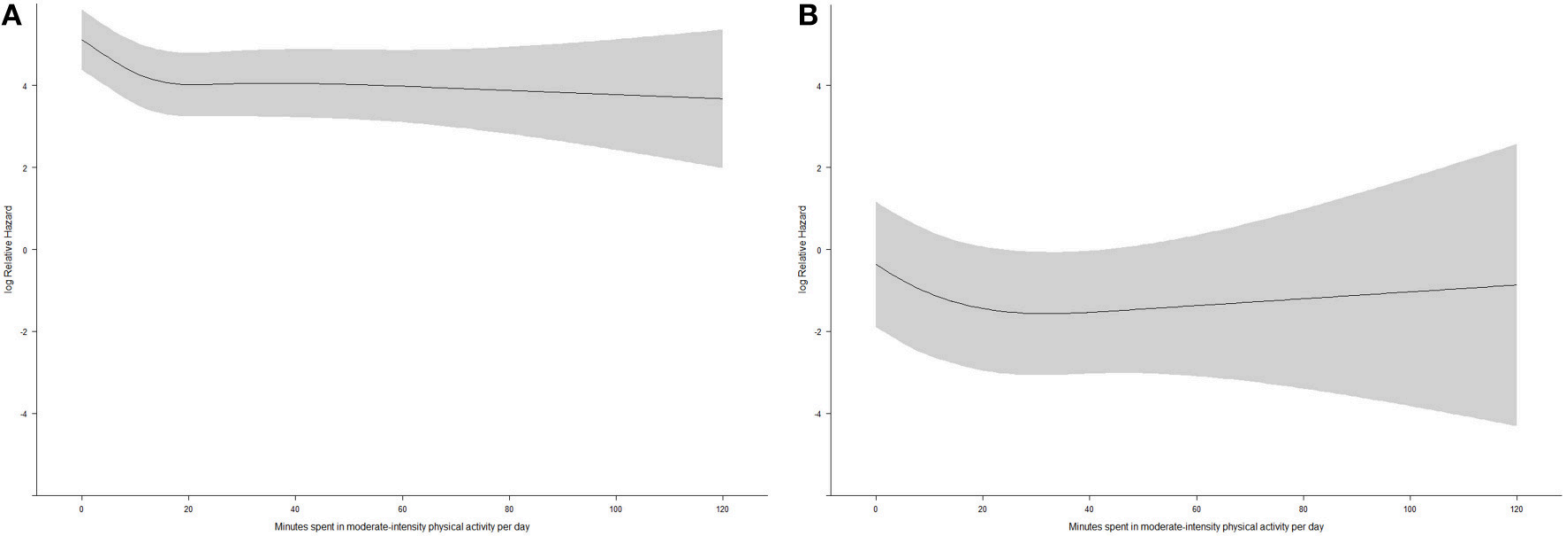
**(A)** The dose-response relationship between time spent in moderate-intensity physical activity and all-cause mortality from restricted cubic spline regression (time spent in sedentary behaviors per day ≤10.9 h, *N* = 5599, shaded area: 95% confidence interval); **(B)** The dose-response relationship between time spent in moderate-intensity physical activity and all-cause mortality from restricted cubic spline regression (time spent in sedentary behaviors per day >10.9 h, *N* = 1407, shaded area: 95% confidence interval).

To examine the interaction effect between moderate physical activity and sedentary behaviors on all-cause mortality, a Cox regression was fitted among participants with time spent in moderate physical activity per day ≤14.1 (Figure 3A) and 14.1 min (Figure 3B), as well as among participants with time spent in sedentary behaviors per day ≤10.9 (Figure 4A) and > 10.9 h (Figure 4B). Figures 3, 4 show that the associations of moderate physical activity and sedentary behaviors on all-cause mortality were independent of each other.

**Figure 5:**
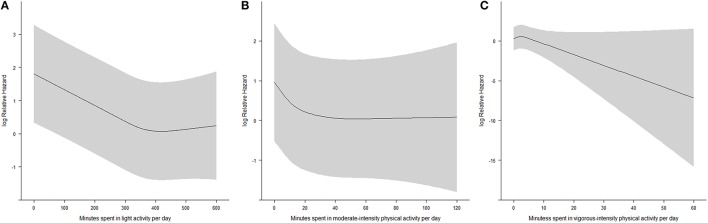
**(A)** The dose-response relationship between daily replacement of 1 min of sedentary time with 1 min spent in light activity and all-cause mortality from restricted cubic spline regression (*N* = 7006, shaded area: 95% confidence interval); **(B)** The dose-response relationship between daily replacement of 1 min of sedentary time with 1 min spent in moderate-intensity physical activity and all-cause mortality from restricted cubic spline regression (*N* = 7006, shaded area: 95% confidence interval); **(C)** The dose-response relationship between daily replacement of 1 min of sedentary time with 1 min spent in vigorous-intensity physical activity and all-cause mortality from restricted cubic spline regression (*N* = 7006, shaded area: 95% confidence interval).

Figure 5 shows that daily replacement of 1 min of sedentary time with 1 min spent in light activity, moderate-intensity physical activity, vigorous-intensity physical activity, and all-cause mortality in the isotemporal substitution model. Figure 5A shows the daily replacement of 1 min of sedentary time with 1 min of light activity demonstrated a reverse J-shaped association with mortality, with the turning point at about 400 min per day. Figure 5B shows that the daily replacement of 1 min of sedentary time with 1 min of moderate-intensity physical activity demonstrated a reverse J-shaped association with mortality, with the turning point at about 15 min per day. Figure 5C shows that the risk of all-cause mortality decreased with time spent in vigorous-intensity physical activity in a linear manner.

**Figure 6:**
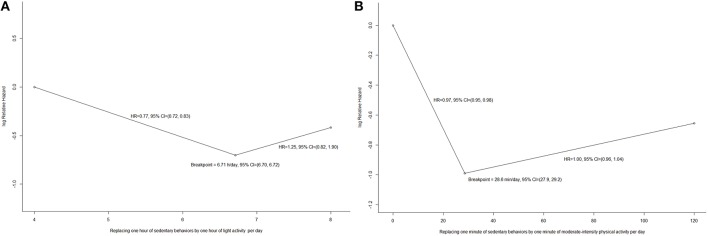
**(A)** The relationship between daily replacement of 1 min of sedentary time with 1 min spent in light activity and all-cause mortality from segmented regression (*N* = 7006); **(B)** The relationship between daily replacement of 1 min of sedentary time with 1 min spent in moderate-intensity physical activity and all-cause mortality from segmented regression (*N* = 7006).

Segmented Cox regression with one breaking point was used to analyze the dose-response relationship between isotemporal substitution of light activity and moderate-intensity physical activity on mortality. Figure 6 shows the log relative hazard yielded from the segmented Cox regression. The fitted breakpoint for the daily replacement of sedentary time with light activity and moderate-intensity physical activity were 6.71 h (SE 0.05) and 28.6 min (SE 0.2), respectively. Each additional replacement of sedentary hour by light activity per day was associated with a HR of 0.77 (95% CI: 0.72, 0.83, *P* < 0.001) among participants who had less than 6.71 h per day of light activity. Among participants who spent at most 28.6 min per day on moderate-intensity physical activity, each additional replacement of sedentary minute by moderate-intensity physical activity per day was associated with a HR of 0.97 (95% CI: 0.95, 0.98, *P* = 0.03), whilst among participants who spent more than 28.6 min per day, each additional replacement minute was not associated with all-cause mortality (HR: 1.00, 95% CI: 0.96, 1.04, *P* = 0.99). Each additional replacement of sedentary minute by vigorous-intensity physical activity per day was associated with an insignificant HR of 0.96 (95% CI: 0.89, 1.03, *P* = 0.25).

## Discussion

This study found that (1) the health benefit of moderate-intensity physical activity is stronger among those spending 15 min or less on such activity per day, which agrees with other research examining the dose-response relationship between physical activity and all-cause mortality (Moore et al., 2012; Arem et al., 2015); (2) there is a threshold of 10.9 h for sedentary behaviors per day, above which there is a positive association with all-cause mortality; and (3) associations of moderate physical activity and sedentary behaviors on all-cause mortality were independent of each other. This result also agrees with other research examining the dose-response relationship between sedentary behaviors and all-cause mortality (Matthews et al., 2015; Pavey et al., 2015). Previous studies examining dose-response relationship treated time spent in physical activity and sedentary behaviors as categorical variables, and here we used segmented Cox regression to empirically determine the break-points of the dose-response relationship. Results of our analysis extended the findings shown among the subgroup of aged ≥50 in NHANES 2003–2006 participants (Schmid et al., 2015, 2016; Fishman et al., 2016). The association of vigorous-intensity physical activity with all-cause mortality was not significant; however, this could be explained by the aforementioned finding that more than half of the sample (*n* = 3776, 53.9%) did not engage in any vigorous-intensity physical activity during the monitoring period, leading to an unstable models with large standard errors.

Among those who spent less than 15 min of moderate-intensity physical activity per day, increasing their engagement to 15 min per day seems to achieve the best marginal gain. Many population-based studies have examined the association of less-than-recommended physical activity volume and all-cause mortality, and similar findings have been obtained (Wen et al., 2011; Moore et al., 2012). The current physical activity recommendation of at least 150 min of moderate-to-vigorous physical activity per week is unrealistic for most healthy adults; as evidenced by the low compliance across the globe (Physical Activity Guidelines Advisory Committee, 2008; Marques et al., 2015) and no evidence of sustainable effect was found despite continual efforts toward physical activity promotion (Sanchez et al., 2015). One of the major barriers to physical activity is lack of time (Trost et al., 2002); therefore, revising recommendations to 15 min of moderate-intensity physical activity per day would be more efficient and practical. In this sample, more than half of the participants (*n* = 3817, 54.5%) accumulated this amount of physical activity, but only one-fourth (*n* = 1850, 26.4%) met the recommendation of 30 min per day. In fact, walking at a speed of 2.5 mph on a firm surface, which equates to the energy expenditure at the level of moderate-intensity physical activity (Ainsworth et al., 2011), one could walk a total distance of 1 km in 15 min. This is achievable for most healthy adults. Studies have also confirmed that walking is negatively associated with mortality (Gregg et al., 2003). Therefore, further research, especially randomized trials, should be conducted to test the feasibility and effectiveness of the promotion of walking. Alternatively, researchers could test the effect of replacing 1 h of sedentary behaviors with light activity (such as house-cleaning or plant-watering), in which the resultant energy expenditure is equivalent to that of walking 15 min (Matthews et al., 2008).

This study revealed that the HR of sedentary behaviors on all-cause mortality was different for those with different levels of sedentary behaviors. A similar finding was obtained in a meta-analysis, which showed that the HR of sitting time for all-cause mortality for self-reported sitting time of 0–3, >3–7, and >7 h per day were 1.00, 1.02, and 1.05, respectively (Chau et al., 2013). Note that we found a higher HR for those with at least 10.8 h of accelerometer-measured sedentary time per day (HR = 1.15). The difference between the volume of sedentary behaviors measured by accelerometer was 3–3.5 h per day more than that with a single-item questionnaire (Clemes et al., 2012). We postulate that the discrepancy between accelerometer-measured and self-reported sedentary behavior was caused by both overestimation of accelerometer data and underestimation of self-report data for two obvious reasons. First, actual sedentary time could be confounded by the accelerometer data handling procedure, where non-wear time of less than 60 consecutive minutes was mistakenly classified as sedentary time. Second, if a participant forgot to remove the accelerometer during sleeping time, sleeping time would be recorded as sedentary time. Nowadays, common models of accelerometers made for measuring physical activity are water-proof (to allow 24-h wearing time) and can detect acceleration from three axes to allow angulation determination, so that classification between non-wear time and sedentary behaviors can be improved. Therefore, we can foresee that future studies based on accelerometer data will produce more accurate results.

The associations between accelerometer-measured physical activity and sedentary behaviors on all-cause mortality found in this study were stronger than those that have been obtained with self-reported physical activity (Samitz et al., 2011) and sedentary behaviors (Pavey et al., 2015). This discrepancy is due to the attenuation effect caused by measurement error (Heavner et al., 2014) in self-reported activity data (Van Poppel et al., 2010; Lee et al., 2011). This argument is supported by the strong association found between physical activity energy expenditure measured with the gold standard (doubly-labeled water) and all-cause mortality (Manini et al., 2006). Therefore, it is very likely that the previously-reported effects of self-reported physical activity and sedentary behaviors on health outcomes were underestimated.

The strength of this study included the large and national sample, results adjusted for a variety of confounders measured with high validity, and the use of segmented regression to obtain the dose-response associations between physical activity and sedentary behaviors and all-cause mortality, which allowed us to determine the breakpoints objectively and calculate the marginal gain of reducing sedentary behaviors and increasing physical activity for people with different sedentary behaviors and physical activity levels, instead of eyeballing a dose-response curve.

There were several limitations in this study and the results should be interpreted with cautions. Due to the small number of deceased participants (*n* = 608, 8.7%), we have not examined cause-specific mortality (which can be done after a longer follow-up period) and subgroup analyses were not conducted. Nevertheless, results of this study were consistent with those of diabetic and older adult subgroups in the NHANES 2003–2004 cohort (Schmid et al., 2015; Loprinzi, 2016). Another limitation of the present study is that the representativeness of the sample was questionable, as only participants who provided valid accelerometer data were included, and their characteristics were different from those who did not provided valid data (Lee et al., 2013; Lee, 2015). In this study, physical activity and sedentary behaviors were defined by a single aggregate value, and their changes over time were unknown. It is possible that the effects of physical activity and sedentary behaviors on all-cause mortality were mediated by poor health status, but this hypothesis could not be examined without multiple follow-up data points. Studies have shown that activities of daily living, leisure-time physical activity, and work-related physical activity have different effects on all-cause mortality (Samitz et al., 2011), as do TV viewing and computer game playing (Katzmarzyk and Lee, 2012; Lee and Wong, 2015), but the nature of the activities could not be classified with accelerometer data alone. Due to space limitation, we have not performed any sensitivity analysis using other common accelerometer count thresholds, such as 760 or 2020 counts per min (Tudor-Locke et al., 2012), as moderate-intensity physical activity. It is possible that for participants with low fitness level, counts per minute of < 1951 could be of moderate-to-vigorous intensity.

In conclusion, this study examined the associations of physical activity and sedentary behaviors on all-cause mortality using accelerometer data, and provided empirical support for recommending at least 15 min per day of moderate-intensity physical activity and limiting sedentary time to 10.9 h per day to reduce the risk of all-cause mortality.

## Author contributions

The author confirms being the sole contributor of this work and approved it for publication.

### Conflict of interest statement

The author declares that the research was conducted in the absence of any commercial or financial relationships that could be construed as a potential conflict of interest.

## References

[B1] Ainsworth B. E. Haskell W. L. Herrmann S. D. Meckes N. Bassett D. R. J. Tudor-Locke C.. (2011). 2011 Compendium of physical activities: a second update of codes and MET values. Med. Sci. Sports Exerc. 43, 1575–1581. 10.1249/MSS.0b013e31821ece12 21681120

[B2] Arem H. Moore S. C. Patel A. Hartge P. De Gonzalez A. B. Visvanathan K.. (2015). Leisure time physical activity and mortality: a detailed pooled analysis of the dose-response relationship. JAMA Intern. Med. 175, 959–967. 10.1001/jamainternmed.2015.0533 25844730 PMC4451435

[B3] Biswas A. Oh P. I. Faulkner G. E. Bajaj R. R. Silver M. A. Mitchell M. S.. (2015). Sedentary time and its association with risk for disease incidence, mortality, and hospitalization in adults. Ann. Intern. Med. 162, 123–132. 10.7326/M14-1651 25599350

[B4] Chau J. Y. Grunseit A. C. Chey T. Stamatakis E. Brown W. J. Matthews C. E.. (2013). Daily sitting time and all-cause mortality: A meta-analysis. PLoS ONE 8:e80000. 10.1371/journal.pone.0080000 24236168 PMC3827429

[B5] Clemes S. A. David B. M. Zhao Y. Han X. Brown W. (2012). Validity of two self-report measures of sitting time. J. Phys. Act. Health 9, 533–539. 21946087 10.1123/jpah.9.4.533

[B6] Durrleman S. Simon R. (2006). Flexible regression models with cubic splines. Stat. Med. 8, 551–561. 10.1002/sim.4780080504 2657958

[B7] Fishman E. I. Steeves J. A. Zipunnikov V. Koster A. Berrigan D. Harris T. A.. (2016). Association between objectively measured physical activity and mortality in NHANES. Med. Sci. Sports Exerc. 48, 1303–1311. 10.1249/mss.0000000000000885 26848889 PMC4911242

[B8] Freedson P. S. Melanson E. Sirard J. R. (1998). Calibration of the computer science and applications, Inc. accelerometer. Med. Sci. Sports Exerc. 30, 777–781. 10.1097/00005768-199805000-00021 9588623

[B9] Gregg E. W. Gerzoff R. B. Caspersen C. J. Williamson D. F. Narayan K. M. V. (2003). Relationship of walking to mortality among US adults with diabetes. Arch. Intern. Med. 163, 1440–1447. 10.1001/archinte.163.12.1440 12824093

[B10] Haskell W. L. Lee I. M. Pate R. R. Powell K. E. Blair S. N. Franklin B. A.. (2007). Physical activity and public health: updated recommendation for adults from the American College of Sports Medicine and the American Heart Association. Circulation 116, 1081–1093. 10.1161/CIRCULATIONAHA.107.185649 17671237

[B11] Heavner K. Newschaffer C. Hertz-Picciotto I. Bennett D. Burstyn I. (2014). Quantifying the potential impact of measurement error in an investigation of autism spectrum disorder (ASD). J. Epidemiol. Community Health 68, 438–445. 10.1136/jech-2013-202982 24470431

[B12] Katzmarzyk P. T. Lee I. M. (2012). Sedentary behaviour and life expectancy in the USA: a cause-deleted life table analysis. BMJ Open 2:e000828. 10.1136/bmjopen-2012-000828 22777603 PMC3400064

[B13] Kozey-Keadle S. Libertine A. Lyden K. Staudenmayer J. Freedson P. S. (2011). Validition of waerable monitors for assessing sedentary behavior. Med. Sci. Sports Exerc. 43, 1561–1567. 10.1249/MSS.0b013e31820ce174 21233777

[B14] Lee P. H. (2015). A sensitivity analysis on the variability in accelerometer data processing for monitoring physical activity. Gait Posture 41, 516–521. 10.1016/j.gaitpost.2014.12.008 25540989

[B15] Lee P. H. Macfarlane D. J. Lam T. H. (2013). Factors associated with participant compliance in studies using accelerometers. Gait Posture 38, 912–917. 10.1016/j.gaitpost.2013.04.018 23688408

[B16] Lee P. H. Macfarlane D. J. Lam T. H. Stewart S. M. (2011). Validity of international physical activity questionnaire short form (IPAQ-SF): a systematic review. Int. J. Behav. Nutr. Phys. Act. 8, 115. 10.1186/1479-5868-8-115 22018588 PMC3214824

[B17] Lee P. H. Wong F. K. Y. (2015). The association between time spent in sedentary behaviors and blood pressure: a systematic review and meta-analysis. Sports Med. 45, 867–880. 10.1007/s40279-015-0322-y 25749843

[B18] Loprinzi P. D. (2016). The effects of objectively-measured, free-living daily ambulatory movement on mortality in a national sample of adults with diabetes. Physiol. Behav. 154, 126–128. 10.1016/j.physbeh.2015.11.022 26626815

[B19] Manini T. M. Everhart J. E. Patel K. V. Schoeller D. A. Colbert L. H. Visser M.. (2006). Daily activity energy expenditure and mortality among older adults. JAMA 296, 171–179. 10.1001/jama.296.2.171 16835422

[B20] Marques A. Sarmento H. Martins J. Saboga Nunes L. (2015). Prevalence of physical activity in European adults - Compliance with the World Health Organization's physical activity guidelines. Prev. Med. 81, 333–338. 10.1016/j.ypmed.2015.09.018 26449407

[B21] Matthews C. E. Chen K. Y. Freedson P. S. Buchowski M. S. Beech B. M. Pate R. R.. (2008). Amount of time spent in sedentary behaviors in the United States, 2003-2004. Am. J. Epidemiol. 167, 875–881. 10.1093/aje/kwm390 18303006 PMC3527832

[B22] Matthews C. E. Moore S. C. Sampson J. Blair A. Xiao Q. Keadle S. K.. (2015). Mortality benefits for replacing sitting time with different physical activities. Med. Sci. Sports Exerc. 47, 1833–1840. 10.1249/MSS.0000000000000621 25628179 PMC4515413

[B23] Mekary R. A. Willett W. C. Hu F. B. Ding E. L. (2009). Isotemporal substitution paradigm for physical activity epidemiology and weight change. Am. J. Epidemiol. 170, 519–527. 10.1093/aje/kwp163 19584129 PMC2733862

[B24] Moore S. C. Patel A. V. Matthews C. E. De Gonzalez A. B. Park Y. Katki H. A.. (2012). Leisure time physical activity of moderate to vigorous intensity and mortality: a large pooled cohort analysis. PLoS Med. 9:e1001335. 10.1371/journal.pmed.1001335 23139642 PMC3491006

[B25] Moshfegh A. J. Baer D. Cleveland L. Rhodes D. Sebastian R. Staples R. (2003). Validation of reported energy intakes in 24-hour dietary recalls using USDA automated multiple-pass method. FASEB J. 17:A281.

[B26] Muggeo V. M. R. (2003). Estimating regression models with unknown break-points. Stat. Med. 22, 3055–3071. 10.1002/sim.1545 12973787

[B27] Muggeo V. M. R. (2008). Segmented: an R Package to fit regression models with Broken-Line relationships. R News 8, 20–25.

[B28] Orrow G. Kinmonth A. L. Sanderson S. Sutton S. (2012). Effectiveness of physical activity promotion based in primary care: systematic review and meta-analysis of randomised controlled trials. BMJ 344:e1389. 10.1136/bmj.e1389 22451477 PMC3312793

[B29] Pavey T. G. Peeters G. Brown W. J. (2015). Sitting-time and 9-year all-cause mortality in older women. Br. J. Sports Med. 49, 95–99. 10.1136/bjsports-2012-091676 23243009

[B30] Physical Activity Guidelines Advisory Committee (2008). Physical Activity Guidelines Advisory Committee Report, 2008. Washington, DC: Department of Health and Human Services.

[B31] Plasqui G. Westerterp K. R. (2007). Physical activity assessment with accelerometers: an evaluation against double labeled water. Obesity 15, 2371–2379. 10.1038/oby.2007.281 17925461

[B32] Samitz G. Egger M. Zwahlen M. (2011). Domains of physical activity and all-cause mortality: systematic review and dose-response meta-analysis of cohort studies. Int. J. Epidemiol. 40, 1382–1400. 10.1093/ije/dyr112 22039197

[B33] Sanchez A. Bully P. Martinez C. Grandes G. (2015). Effectiveness of physical activity promotion interventions in primary care: a review of reviews. Prev. Med. 76, S56–S67. 10.1016/j.ypmed.2014.09.012 25263343

[B34] Schmid D. Ricci C. Baumeister S. E. Leitzmann M. F. (2016). Replacing sedentary time with physical activity in relation to mortality. Med. Sci. Sports Exerc. 48, 1312–1319. 10.1249/mss.0000000000000913 26918559

[B35] Schmid D. Ricci C. Leitzmann M. F. (2015). Associations of objectively assessed physical activity and sedentary time with all-cause mortality in US adults: the NHANES Study. PLoS ONE 10:e0119591. 10.1371/journal.pone.0119591 25768112 PMC4358950

[B36] Sedentary Behaviour Research Network (2012). Letter to the editor: standardized use of the terms “sedentary” and “sedentary behaviours”. Appl. Physiol. Nutr. Metab. 37, 540–542. 10.1139/h2012-024 22540258

[B37] Trost S. G. Owen N. Bauman A. E. Sallis J. F. Brown W. (2002). Correlates of adults' participation in physical activity: review and update. Med. Sci. Sports Exerc. 34, 1996–2001. 10.1097/00005768-200212000-00020 12471307

[B38] Tudor-Locke C. Camhi S. M. Troiano R. P. (2012). A catalog of rules, variables, and definitions applied to accelerometer data in the National Health and Nutrition Examination Survey, 2003-2006. Prev. Chronic Dis. 9:E113. 10.5888/pcd9.110332 22698174 PMC3457743

[B39] Van Poppel M. N. M. Chinapaw M. J. M. Mokkink L. B. Van Mechelen W. Terwee C. B. (2010). Physical activity questionnaires for adults: a systematic review of measurement properties. Sports Med. 40, 565–600. 10.2165/11531930-000000000-00000 20545381

[B40] Wen C. P. Wai J. P. M. Tsai M. K. Yang Y. C. Cheng T. Y. D. Lee M. C.. (2011). Minimum amount of physical activity for reduced mortality and extended life expectancy: a prospective cohort study. Lancet 378, 1244–1253. 10.1016/S0140-6736(11)60749-6 21846575

